# Women’s sexual health improvement: sexual quality of life and pelvic floor muscle assessment in asymptomatic women

**DOI:** 10.3389/fmed.2024.1289418

**Published:** 2024-02-21

**Authors:** Ewa Baszak-Radomańska, Jadwiga Wańczyk-Baszak, Tomasz Paszkowski

**Affiliations:** ^1^Terpa Clinic, OB/GYN Department, Lublin, Poland; ^2^Department of Gynecology, Medical University of Lublin, Lublin, Poland

**Keywords:** gynecology follow-up, pelvic examination, pelvic floor muscle dysfunction, sexual health, sexual quality of life, woman health

## Abstract

**Introduction:**

Problems related to the quality of sexual life in gynecological practice are usually neglected. This study aimed to highlight the significance of this area of concern and evaluate the usefulness of tools, such as patient-reported outcomes (PROs) and pelvic floor examination, to improve women’s sexual wellbeing and to identify predictors of poor quality of sexual life during the well-woman annual visit.

**Methods:**

A cross-sectional study was designed to examine 300 healthy women to determine whether the sexual quality of life (SQOL) questionnaire (on electronic devices) and pelvic floor muscle assessment (the vulva, anus, muscles, and periurethral (VAMP) protocol) of asymptomatic women during the annual bimanual examination (BME) help differentiate patients who would benefit from discussing sexual problems with a gynecologist. Dyspareunia was an exclusion criterion.

**Results:**

The majority of subjects experienced high sexual wellbeing (82.0% with SQOL score of ≥84), with a mean of 85.7 points. SQOL scores were lower for psychiatric disorders or symptoms (37.0% of subjects), although they did not correlate with age, BMI, parity, contraception use, history of vulvovaginal symptoms, neurosurgical/orthopedic problems, and rectal, bowel, or bladder symptoms. Patients with dyspareunia (16.0% of participants, although they denied it during the face-to-face consultation) had a 3.6 times higher prevalence of low or moderate quality of sexual life. The VAMP protocol score was low in asymptomatic women, 33.0% met positive criteria (VAMP+, NRS ≥3) for pelvic floor dysfunction (overactivity), although at borderline levels. VAMP+ was positively correlated with chronic pain and genitourinary symptoms, but neither with dyspareunia nor incontinence, and was unrelated to the SQOL score (*p* = 0.151).

**Conclusion:**

Women’s sexual health is a global health priority. Finding a way to start a discussion with an asymptomatic patient is crucial to increasing patients’ interest in disclosing a sexual health problem to be resolved. PROs or simple questions about sexual wellbeing direct the discussion mainly toward the at-risk group for sexual deterioration: those with mental health problems and women with dyspareunia. Dyspareunia is considered a predictor of decreased quality of sexual life, a major sexual disorder that should not be overlooked. Gynecological consultation should resolve concerns, identify the problem, and refer for professional sexual care if still needed.

## Introduction

The American College of Obstetricians and Gynecologists (ACOG) issued a Committee Opinion stating that “obstetricians/gynecologists are in a unique position to open a dialogue on sexual health issues” (1). ACOG acknowledged sexual health as an important element of overall health and also supported the World Health Organization’s (WHO) definition of sexual health as “a state of physical, emotional, mental and social well-being in relation to sexuality; it is not merely the absence of disease, dysfunction, or infirmity” (2). “High importance of sexual health to quality of life was reported by 62.2% of men and 42.8% of women” (3).

Understanding the growing importance of sexual health to quality of life, perceived as a sexual quality of life, “is needed to help guide future research efforts, including the development of interventions to enhance patient-provider communication about sexual concerns associated with common conditions and their treatments” (3).

Obstetricians and gynecologists conduct about half of all visits as preventive care for women of reproductive age (4) and provide information on sex as a source of problems in the context of pregnancy or STDs, but sex is a valuable, meaningful activity by which we exist as a species, a source of happiness, and a sign of individual health. Efforts should be made to make doctors feel comfortable talking to patients about sexual health. In the coming era of artificial intelligence in medical care, it is important to broaden the focus during asymptomatic women’s visits to add the value of direct patient–gynecologist medical consultation.

According to current healthcare requirements, “periodic visits for reproductive and well-woman care are recommended, even if individual components of that visit, as cervical cancer screening may not be indicated each year” (1).

Women’s sexual wellbeing problems occur rather frequently; “43% of American women report experiencing sexual problems, with 12% considering this problem leading to personal distress what[*sic*] becomes crucial for sexual dysfunction diagnosis” (5). Sexual dysfunction, according to the International Classification of Diseases, 11th Revision (ICD-11), falls into three main groups, namely, hypoactive sexual desire dysfunction, sexual arousal dysfunctions, orgasmic dysfunctions, and a separate group of sexual pain disorders (6). Some sexual problems can be solved by the patient alone or with a partner, and others still require the help of a gynecologist or other health professional support.

According to the literature, a quarter (26.6%) of community survey participants reported seeking professional help for sexual difficulties. Only 1 in 10 (10.4%) had sought sexual consultation from a gynecologist or urologist (mostly male patients) (7), partly because sexual health issue is usually neglected during routine gynecological well-woman visits (8).

Health professionals revealed obstacles to discussing sexual wellbeing during regular visits with their patients (9–11):

1. Feelings of discomfort and embarrassment due to a lack of adequate knowledge.
2. Difficulty in referring the patient to a sex therapist, a psychologist with experience in sexual health, sex educators (long waiting list and consultation fee not covered by insurance), or even they are unavailable in the national healthcare system.
3. Patients are focused on other aspects of the follow-up visit, rather on gynecological symptoms, cervical cancer prevention, or contraception.
4. There is not enough time to discuss sexual problems. The patient spends limited time per visit and is not prepared to extend it, and there is a lack of payment for lengthy discussions with the patient.
5. Fear of offending patients, especially the elderly and people of the opposite sex.
6. Underestimation of the prevalence of sexual problems.

On the other hand, patients are also uncomfortable discussing sexual health issues because of a lack of privacy (nurse attendance) during the visit. Patients do not want to embarrass the health professional by asking intimate questions and are not convinced that the doctor has enough knowledge about sexual problems. Additionally, patients and their partners have other sources to derive information on sexual education, such as the Internet, their surroundings (family and peer group), and healthcare professionals who may not be an authority on sexual health (9, 10).

An effort should be made to change the attitude on both sides. Appreciating the patient’s sexual wellbeing, assessing the risk group for sexual problems, and the art of asking questions to start the conversation would be crucial to supporting the patient. According to ACOG’s Committee Opinion, “Obstetrician–gynecologists should initiate a clinical discussion of sexual function during routine care visits to identify issues that may require further exploration and to help destigmatize discussion of sexual function for patients” (11).

Because of the lack of capability to discuss sexual wellbeing with every asymptomatic woman, finding the risk group for sexual dysfunction seems crucial. Assessing the quality of sexual life in asymptomatic women using validated tests is considered in the literature as a useful tool for differentiating patients who will benefit from further investigation and management. The sexual quality of life (SQOL) questionnaire for female patients was employed to assess “the quality of sexual life, the impact of possible sexual dysfunction on the sexual well-being in self-esteem, emotional and relationship aspects” (12).

Detailed assessment tools in the form of self-report questionnaires patient-reported outcomes (PROs) and diagnostic tests to objectify sexual dysfunction rather than waiting for a spontaneous admission of sexual problems can be used in gynecology, or at least in special circumstances, in a risk group, or when a problem is suspected.

In clinical practice, simple screening questions, a comprehensive history, inquiries about contraception, followed by an appropriate bimanual examination (BME) including vulvar and anal visualization, with speculum examination and a cervical cancer screening if appropriate (in a 3-year interval, when negative), and a bimanual pelvic exam (BPE) is undertaken to exclude pelvic inflammatory disease and pelvic mass. A breast examination is also performed. This is all that meets the gynecological and sexual health requirements of asymptomatic women. The advantages of BME include the detection of precancerous conditions or the early stages of gynecologic cancers, vulvar skin lesions, and foreign bodies when women are unaware of symptoms (13). Fifty-two million BPEs were conducted in the United States in 2015 (14), which is a great opportunity to gather as much information as possible about a woman’s health and quality of life.

In women with sexual dysfunction, the scope of the examination is routinely extended to pelvic floor muscles, and a physical examination of the clitoris is undertaken. If the glans corona is not fully visible, clitoral adhesions should be suspected, which “has been found in up to 22% of women seeking evaluation for sexual dysfunction” (15).

Overactive pelvic floor muscles are associated with superficial dyspareunia (which is caused mainly by provoked vulvodynia), while pelvic inflammatory disease, endometriosis, pelvic venous disorders (PeVDs), and pelvic mass are mainly responsible for deep dyspareunia (16).

Female sexual dysfunction should be treated as a multifactorial and complex concern for a woman’s health. Pelvic floor muscles are also responsible for sexual function (arousal, sensation, penetration, and orgasm). Pelvic floor dysfunction in women includes a wide range of clinical disorders: “urinary incontinence (55.8% in Spain women population), fecal incontinence (in 10.4%), symptomatic uterine prolapse (in 14.0%), and pelvic-perineal region pain syndrome (18.7%)” (17). Urinary incontinence (stress, urge, or mixed, functional incontinence, and overflow incontinence) is defined as involuntary leakage of urine (18). Pelvic floor muscle dysfunction can be presented as a hypotonic state (or decreased tone) and primarily as an overactivity (increased tone), according to the recent guideline “An International Continence Society (ICS) report on the terminology for pelvic floor muscle assessment” classification described as “Disorder of increased PFM tone,” divided by pelvic floor tension myalgia, pelvic floor myofascial syndrome, and also as “Disorder of PFM pain” where pelvic floor myalgia or even disorder of decreased PFM tone should be differentiated (19). More precisely, the term “pelvic floor muscle overactivity” is used. “Pelvic floor overactivity is a condition that pelvic floor muscles are not relaxing or even contract, when relaxation is functionally needed, during micturition or defecation. Woman[*sic*] with PFMD/overactivity usually present with a combination of symptoms: gastrointestinal, gynecological, musculoskeletal, sexual, and urological comorbidities, which may also reflect peripheral and central sensitization (CS) mechanisms, emotional and psychological states” (20, 21) and comorbid functional (or nociplastic) pain (20, 21). Although some protocols are suggested (22, 23), for example, “Checklist of PFM clinical assessment, applicable to signs and investigations, External assessment per perineum: Visual observation or Digital palpation, Tests of digital palpation per vaginam/per rectum” (19), there are no standardized protocols for clinical practice (22–24). The systematic review did not demonstrate the superiority of any diagnostic test over a predefined reference test for PFM overactivity, and there is “a lack of a[*sic*] validated diagnostic criteria which must be addressed to progress with meaningful research in this field” (22, 23).

Because there is no simple tool or standardized procedure for pelvic floor overactive muscle examination, the study authors developed the vulva, anus, muscles, and periurethral (VAMP) protocol for PFM overactivity state confirmation or exclusion (25, 26).

Pelvic floor evaluation is indicated in patients with vulvar pain or discomfort, provoked or spontaneous (e.g., perceived “recurrent intimate infections”), or in women with chronic pelvic pain (CPP). Considering the relationship between pelvic floor muscles and dyspareunia, it is important to emphasize that “after controlling for distress levels, sexual pain was the most frequent sexual problem reported by lesbians and heterosexual women” (27). The study hypothesis is the usefulness of the bimanual pelvic exam (BPE) focusing on pelvic muscle assessment in asymptomatic patients to reveal PFM overactivity as a predisposition to dyspareunia, chronic vulvar pain, or silent complaints, which would be useful for further sexual wellbeing discussions with the gynecologist.

Since no single laboratory test is recommended as a marker of sexual dysfunction, further management is dictated by clinical evaluation (28). When analyzing other indirect tools that could be used to facilitate communication about sexual problems with asymptomatic patients, the SQOL Questionnaires and the condition of the pelvic floor muscles are considered.

### Aim of the study

The purpose of this cross-sectional study was to highlight the interest in sexual health problems during a well-woman gynecological visit and to evaluate the usefulness of tools, such as patient-reported outcomes (PROs) and pelvic floor examination, to improve women’s sexual wellbeing and to identify predictors of poor quality of sexual life.

## Materials and methods

### Participants

Three hundred asymptomatic, generally healthy women were included in the study during routine gynecological visits in the outpatient Terpa clinic in Lublin, Poland. The center focused mainly on vulvar diseases. The participants included were non-pregnant, adult, premenopausal women between 18 and 50 years old. Well-women visits of study participants took place between 17 January 2022 and 28 April 2023. All patients were examined by the study authors. The size of the study group was contingent on obtaining sufficient statistical power from the results obtained.

Patients with serious general diseases, gynecological diseases requiring treatment, pelvic organ prolapse, vulvovaginal disease, and dyspareunia were excluded from the study.

### Instruments

Patients who met the study’s inclusion criteria were asked to consent to participate in the study and were asked by their gynecologist whether they would like to talk about sexual health.

Vital signs (weight and height, pulse, and blood pressure) were taken, and body mass index (BMI) was calculated.

Well-women were interviewed using electronic devices with online data collection (when a gynecological visit was completed) about their age, health (mental, pain, vulvar., sexual, general, and surgery), dysuria and urine incontinence, bowel and anal symptoms, contraception, parity, sexual relationship and orientation, educational level and residency, and SQOL questionnaires were fulfilled (patient-reported outcomes, PROs) and measured. The sexual quality of life - female (SQOL) questionnaire, designed by Symonds (12), “consists of 18 items, and each item is rated on a six-point response category (1 - completely agree to 6 - completely disagree). The scores on this scale range from 18 to 108, and a higher score indicates better sexual quality of life.” The following formula was used for the score calculation:


$$\begin{array}{l} \mathrm{Scale}\;\mathrm{score}=\\ \frac{\mathrm{The}\;\mathrm{sum}\;\mathrm{of}\;\mathrm{components}-\mathrm{The}\;\mathrm{lowest}\;\mathrm{possible}\;\mathrm{score}\;}{\mathrm{Possible}\;\mathrm{raw}\;\mathrm{score}\;\mathrm{range}}\;x\;100 \end{array}$$

It is a “self-reported instrument and includes four subscales (domains). Examples of items are provided in parentheses: psychosexual feelings (measuring anger, worry of partner’s hurt or rejection), sexual and relationship satisfaction (enjoy, good feeling about oneself), self-worthlessness (feeling like less of a woman, feeling of guilt), and sexual repression (loss of pleasure, avoiding). The measure is a useful instrument to assess female quality of sexual life, with categorical ratings of poor (score range < 51), moderate (score range from 52 to 84), and good (score > 84)” (29). According to the authors’ assumptions of the questionnaire, the results were calculated as an overall total score and not as separate domains.

The SQOL questionnaire was chosen for this study because it does not in itself impose sexual problems. A Polish version of the SQOL questionnaire has been used (30, 31).

During the follow-up visit, a bimanual examination (BME) was performed, which focused on sexual health. A thorough visualization of the clitoris and the whole vulvar area was conducted to evaluate the visible source of vulvar pain, itchiness, or discomfort and to rule out any clitoral adhesions. Any visible or detectable vulvovaginal pathology was excluded from the study group.

In the lithotomy position, pelvic floor muscle assessments were performed according to the VAMP protocol (developed by the study authors) (25, 26) based on a two-center study (32, 33).

**Figure 1:**
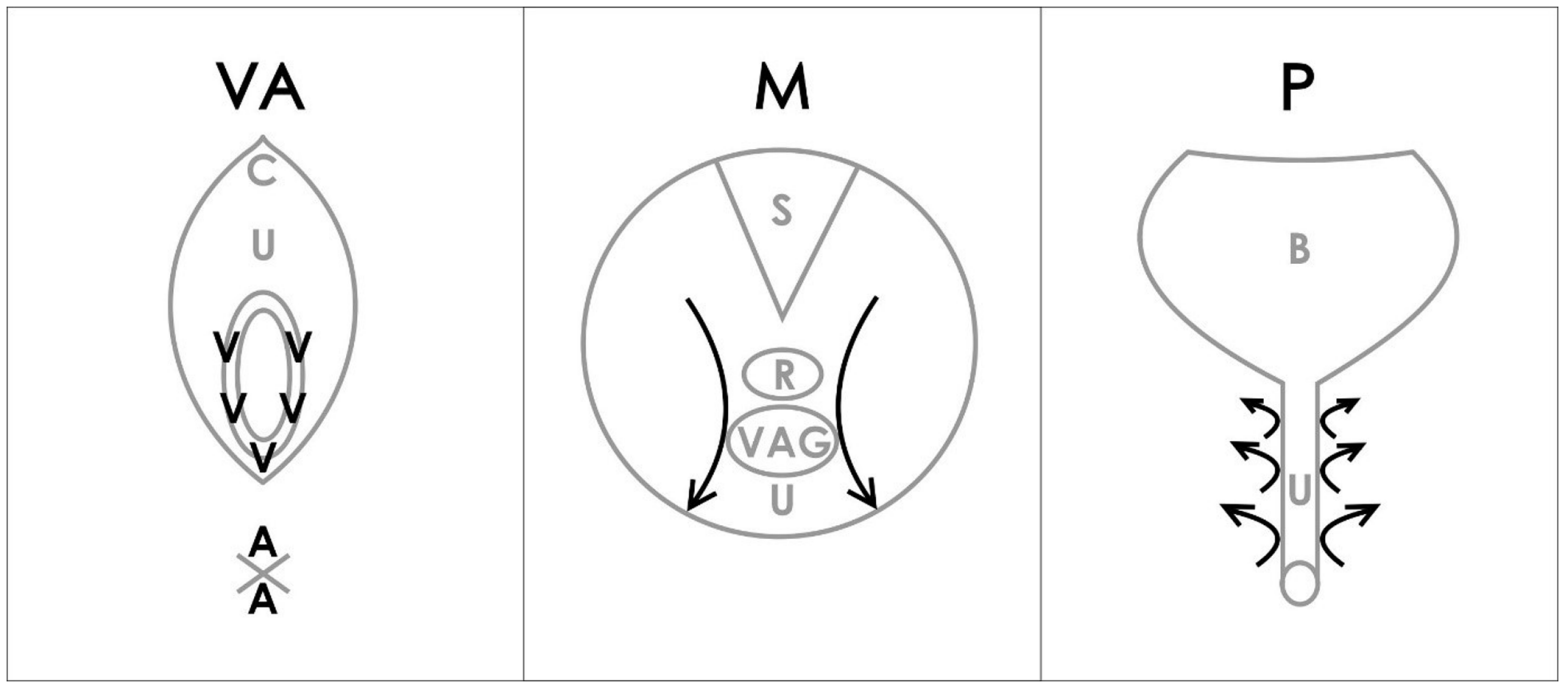
VAMP examination diagram (26). Reproduced with the permission of the Polish Society of Gynecologists and Obstetricians. V-vulva: 5 points cotton swab pressure; A-anus: 2 points cotton swab pressure; C-clitoris; U-urethra; M-muscles; S-sacrum; R-rectum; VAG-vagina; P-paraurethral area; B-bladder; arrows-direction of internal digital examination.

During BME, “4 anatomical regions were assessed: the vulva (V) and anus (A) with a cotton swab test, the internal pelvic muscles (M) with a digital examination of the levator ani muscle, and the paraurethral (P) area with digital pressure” (25, 26) (Figure 1). The Numerical Pain Rating Scale (NRS) from 0 to 10 was explained to the patient. Patients were asked to rate each examined point if painful.

The following steps were carried out according to the VAMP protocol (as outlined in Figure 1), described by the study authors in the previous publications (25, 26):

- The Numerical Pain Rating Scale (NRS) was explained, through which the patients were asked to rate each examined point if painful.
- Verbal consent should be taken after an explanation.
- A cotton swab test was performed by applying gentle pressure to five points within the vestibular base of the hymenal remnant (following a vestibular clock from the two to ten position) using a dry swab. The pressure was adjusted to a level tolerated by the patient. Only the maximum NRS rating was noted (V).
- A similar cotton swab test was then used for two points around the anus, with similar pressure as that used for the vulva, and the NRS maximum rating was noted (A).
- The insertion of one lubricated, gloved index finger, for a bimanual transvaginal or rectal examination was performed.
- The palmar side of the index finger was rotated backward to palpate the pelvic floor muscles. The index finger then examines the PFM (vaginally or rectally). Laterally, progressing from the posterior section, on each side of the rectum (from as far back as possible), a bilateral sliding motion was performed, with marked pressure applied to the muscles along the iliococcygeus muscle to the anterior portion of the puborectalis muscle (mid-muscle belly technique), but avoiding the rectum. This is repeated bilaterally with marked tension applied to muscles but within the acceptable pain threshold of individual patients. This allows for the assessment and differentiation of pain severity in superficial PFM (bulbospongiosus, ischiocavernosus, painful external muscles on pressure during palparion) and deep PFM (levator ani) until the patient’s accepted pain threshold is reached. The maximum NRS rating was noted (M) in the medical records.
- For paraurethral area examination, the palmar side of the index finger was rotated upward, laterally to the urethra, and similarly to the point of urethra detachment from the pubic bone. The movement was performed from the outside to the inside of the pubis, lengthwise, on both the right and left sides. The pressure was increased with particular attention to patient pain tolerance, and the maximum NRS rating was noted (P).

In every patient, the VAMP score was determined. The physical exam results were recorded under the VAMP acronym (e.g., VAMP 6068), which reflects the maximum NRS ratings in the four areas: the vulva, anus, muscles, and paraurethra. Based on previous publications, “the VAMP score cut-off (NRS on pressure) of consecutive features: V, score 2; A, not relevant; M, score 3; and P, score 3, was established” (25, 26). The VAMP scores were previously evaluated in patients with vulvodynia and in the pain-free control group in various studies (25, 26). In clinical practice, if any VAMP score was ≥3, pelvic floor muscle overactive dysfunction was diagnosed (25, 26).

### Procedure

All participants were informed of the objectives, characteristics, and procedures of the study and signed the informed consent form prior to the bimanual examination and completing the questionnaire. This research follows the requirements and protocols of the study, approved by the appropriate institutional review board (IRB) (Bioethics Committee at the Medical University of Lublin, KE-0254/210/2021).

### Statistical analysis

The results obtained were subjected to statistical analysis. The values of the quantitative variables analyzed were presented using the mean, median, lower and upper quartiles, and standard deviation, and the qualitative variables were presented using count and percentage.

The correlation of qualitative variables was assessed using the Chi-square test. The verification of the normality of the distribution of variables in the study groups was performed using the Shapiro–Wilk normality test. Student’s *t*-test was applied to examine differences between the two groups, and the Mann–Whitney test was used if the conditions for its use were not met. To evaluate differences between three or more groups, the Kruskal–Wallis test was used. Pearson’s correlation was used to check the relationship between some variables.

Multivariate logistic regression analysis was used to estimate the risk of poorer quality of sexual life based on selected variables. A significance level of a value of *p* < 0.05 was assumed.

The data were collected on electronic devices, and preliminary statistical analyses were performed in the statistical software.^1^ The advanced analysis was performed using Statistica 9.1 (StatSoft, Poland) and PQStat 1.8.2 software.

## Results

All women agreed to participate in the study when asked by a gynecologist.

Every woman was interested in a sexual health discussion with a gynecologist on an annual follow-up visit (*N* = 300; 100%). However, 16% of the participants (*N* = 48) were untrue and denied sexual pain or other discomfort when asked by a gynecologist (exclusion criteria). Non-sexually active participants (*N* = 36; 12%) were included in the study analysis. Table 1 shows the sociodemographic and additional characteristics of the participating cohort.

**Table 1:** Characteristics of the study population (*N* = 300).

Characteristics	No	%
Age: years mean (S/D; range)	34 (8.7; 18–50)	–
BMI (kg/m^2^): mean (S/D; range)	23.3 (4.1; 16.1–41.4)	–
Blood pressure: systolic mean mmHg (S/D; range mmHg)	125 (13.6; 89–167)	–
Blood pressure: diastolic mean mmHg (S/D; range mmHg)	81 (8.9; 55–108)	–
*Having a regular sexual partner*		
Yes	264	88.0
No	36	12.0
Heterosexual	264	88.0
Homosexual	0	0
*Education*		
Primary	12	4.0
Secondary	59	19.7
Tertiary (high school)	229	76.3
*Residency*		
Rural area	74	24.0
Urban area	226	76.0
*Deliveries*		
Yes	162	54.0
No	138	46.0
Natural deliveries: mean (range)	1.0 (0–4)	–
Cesarean: mean (range)	0.89 (0–3)	–
*Dyspareunia (at least 1 episode in the last month) during or after penetration*		
There was no penetration	6	2.0
Yes (although patients were inquired before they were included in the study and it was denied)	48	16.0
No	246	82.0
Contraception method in the last 1 month: No need for contraception (no penile penetration or intention to get pregnant)	23	7.7
Yes	246	82.0
No (with no intention to get pregnant)	31	10.3
*Vulvovaginal symptoms in the last 1 year*		
No	230	76.7
1–3 times	72	20.7
4 times and more	8	2.7
*Chronic pain symptoms in the last 1 year (low back or joint pain, migraine, temporomandibular junction dysfunction, and gastric symptoms)*		
Yes	246	82.0
No	54	18.0
*Neurosurgical and orthopedic injuries or surgeries*		
Yes	25	8.3
No	275	91.7
*Anal and bowel symptoms*		
Yes	31	10.3
No	269	89.7
*Bladder symptoms*		
Yes	62	21.7
No	238	79.3
*Voiding episodes per day*		
Up to 10 times	275	91.6
More than 10 times	25	8.4
*Urinary incontinence*		
Yes	93	31.0
Stress	71	23.7
Urge	12	4.0
Mixed	10	3.3
No	207	69.0
*Psychiatric disorders or symptoms (depression, anxiety, neurosis, and others)*		
Yes	111	37.0
No	189	63.0
*Sleep disturbances (insomnia)*		
Yes	72	24.0
No	228	76.0
*Vulvar pain or other discomfort in the last 3 months (sustained minimum few days)*		
Yes	51	27.0
No	249	83.0

BMI, body mass index; SD, standard deviation.

Almost all participants (*N* = 288; 96%) held bachelor’s degrees or higher, and the vast majority of participants lived in urban areas (*N* = 226; 76%).

### PROs: SQOL results

The mean value of the general quality of life index in relation to sex life for all 300 surveyed women was 85.7 points (SD = 16.3). The highest rating was 100 points, and the lowest was 0 points. The variation in results (range: lowest quadrille 82.2 and highest quadrille 96.7, where 50% results were included) was found to be quite low.

**Figure 2:**
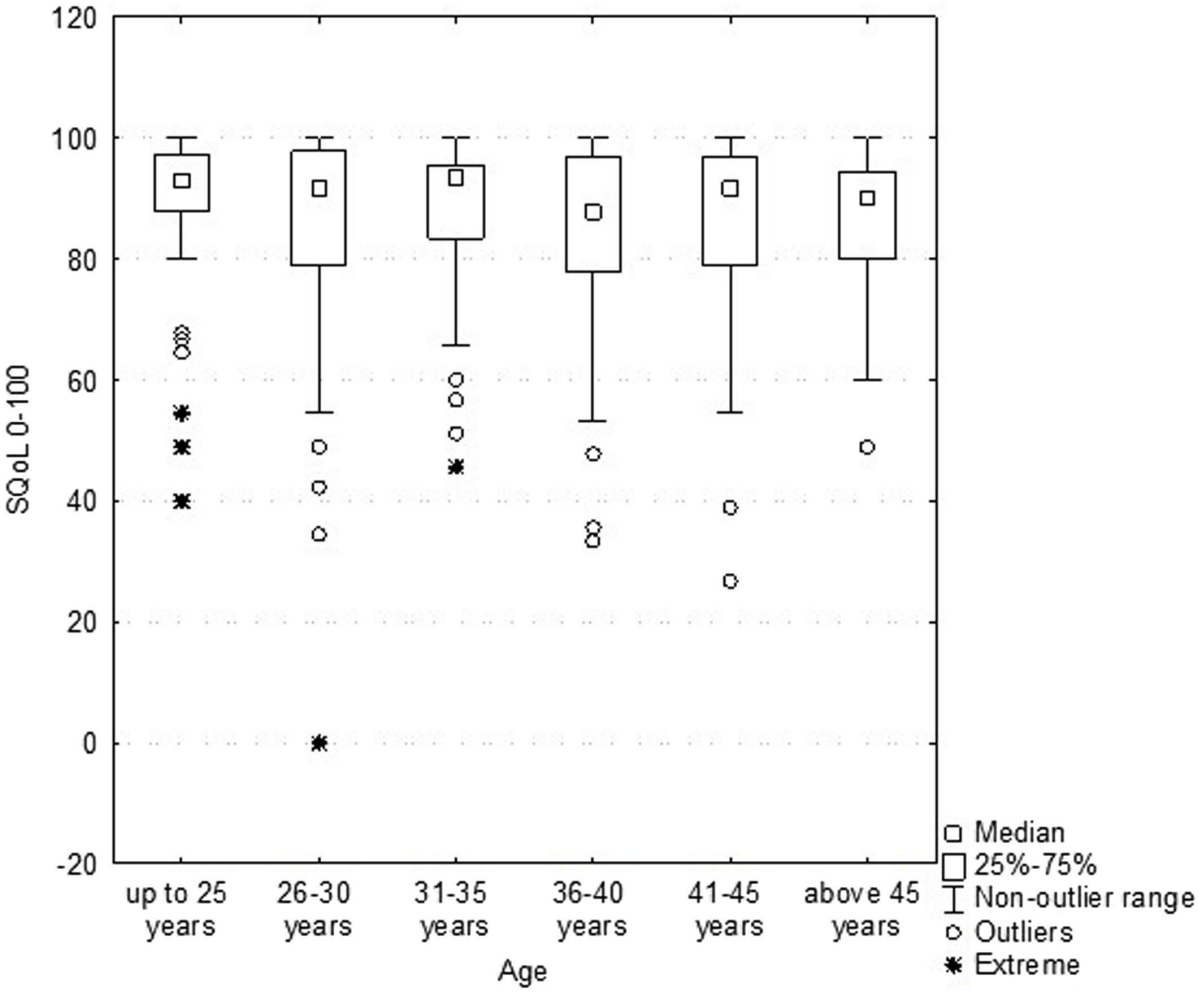
Dependence sexual quality of life (SQOL) score on the women’s age (no statistical correlation).

**Figure 3:**
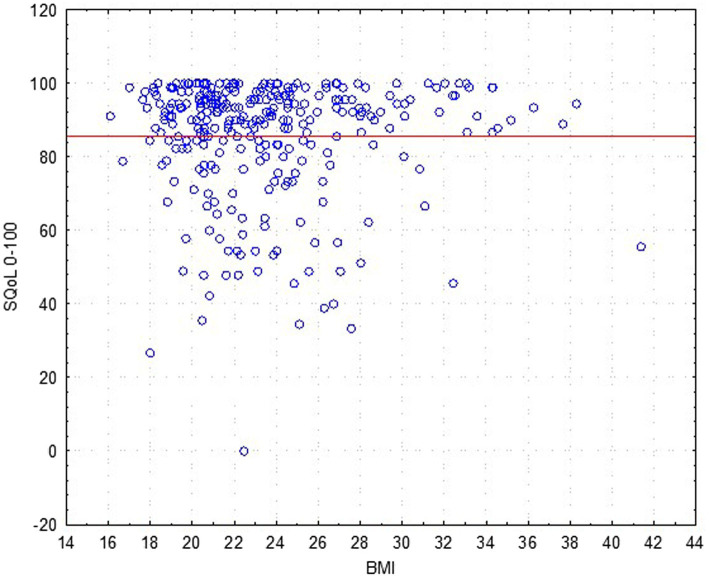
Dependence sexual quality of life (SQOL) score on the women’s body mass index (BMI) (no statistical correlation).

The results revealed that age (Figure 2) and BMI (Figure 3) have no influence on the SQOL in generally healthy women.

An analysis for discriminant validity through a Mann–Whitney test revealed that women with normal weight (BMI ≤ 25.0; *N* = 218; mean rank = 85.9) did not have a significant difference in sexual wellbeing as compared to the overweight (and obesity) group (BMI > 25.0; *N* = 82; mean rank = 84.8), with *Z* = −0.051; *p* = 0.958.

The mean SQOL score was lower in the psychiatric disorders or symptoms (such as anxiety and depressive mood) group (37%; *M* = 81.3; Me = 90.0) than in others (63%; *M* = 88.3; Me = 92.2) (*p* = 0.001). There is no other correlation according to the other six characteristics (Table 2).

**Table 2:** Dependence sexual quality of life (SQOL) score on variables (no statistical correlation).

Variables	Mann–Whitney test: *p*
Parity	*p* = 0.560
Contraception method (non-medical and required medical consultation)	*p* = 0.990
Vulvovaginal symptoms (at least 1 episode/year)	*p* = 0.502
Neurosurgical and orthopedic injuries or surgeries	*p* = 0.849
Anal and bowel symptoms	*p* = 0.569
Bladder symptoms	*p* = 0.615
Psychiatric disorders or symptoms	*p* = 0.001^*^

^*^Dependent variable: sexual quality of life (SQOL). ^**^As significance level of a value of *p* < 0.05.

**Figure 4:**
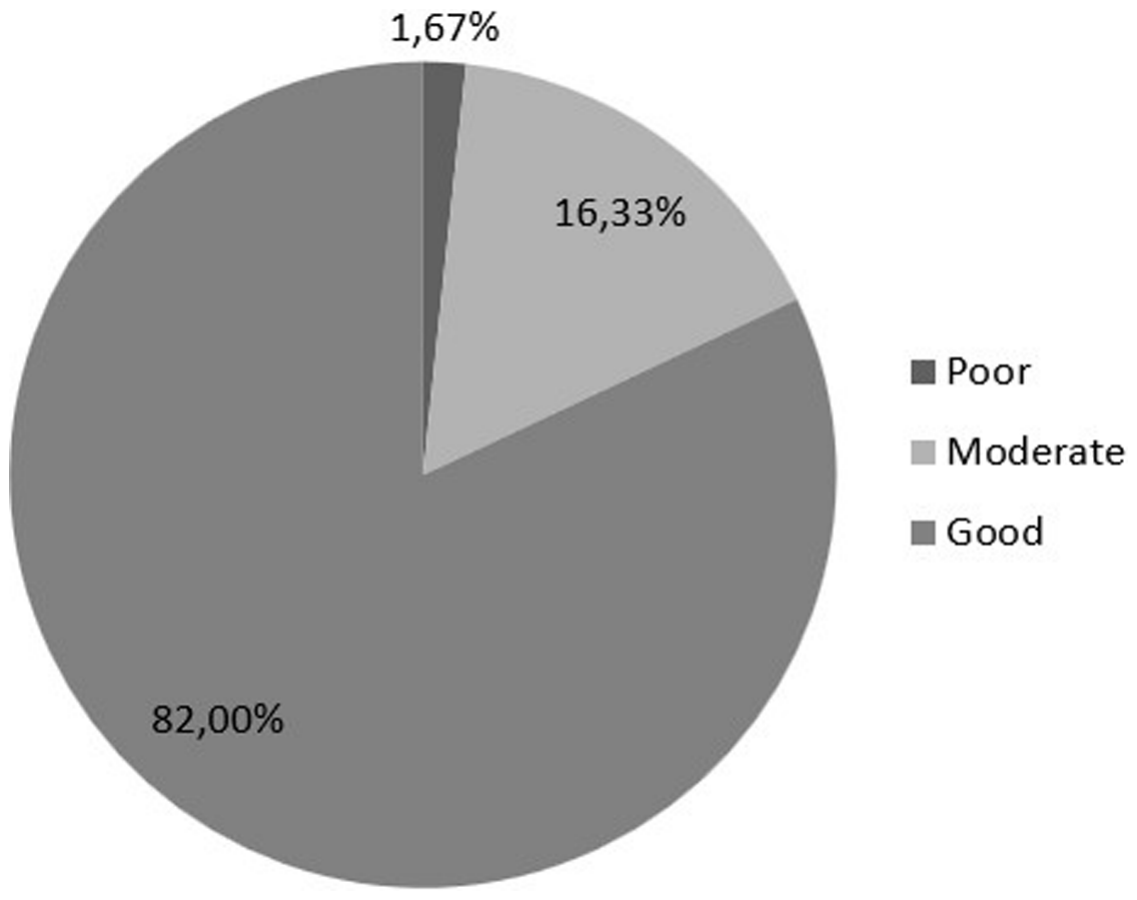
Sexual quality of life (SQOL) score is divided into poor (0–51 points), moderate (52–84 points), and good (85–100 points).

Considering the cutoff scores, the women’s quality of sexual life considered poor/moderate (SQOL score up to 84) was 18% (*N* = 54), and the majority of participants reported their SQOL as high (*N* = 246; 82%). The cohort grouping of poor/moderate/good is presented in Figure 4.

Having a regular sexual partner was declared by 88% of participants (*N* = 264), furthermore, 98% (*N* = 294) have been sexually active with their partner or have masturbated (with vaginal penetration or not).

Logistic regression showed that neither chronic pain symptoms, psychiatric diseases nor urinary incontinence were associated with an evident chance of having a significantly lower SQOL score. The only factor affecting sexual functioning (defined as a poor or moderate level of SQOL, score < 84) was dyspareunia (*p* < 0.05). This was seen in sexually active women who were divided into two groups, namely, no pain during sexual activity and dyspareunia (pain or other discomfort connected with vaginal penetration or with non-penetrative sex). Patients with dyspareunia had a 3.679 times higher chance of having poor or moderate sexual life (in a 95% confidence interval of 1.487–9.102) when compared with non-dyspareunia women. Factors influencing the poor/moderate SQOL based on logistic regression analysis are presented in Table 3.

**Table 3:** Factors influencing the poor/moderate sexual quality of life (SQOL score of <84) based on logistic regression analysis.

Variables	OR (95% CI)	*p*
Chronic pain	6.763 (0.850–53.787)	0.071
Psychiatric disorders or symptoms	1.467 (0.592–3.632)	0.408
Urinary incontinence	2.446 (0.991–6.038)	0.052
Dyspareunia	3.679 (1.487–9.102)	0.005^**^
Likelihood ratio test value of *p*: ^**^p < 0.01		
Pseudo *R*^2^ = 0.136		
*p*_Hosmer–Lemeshow test_ = 0.934		

^*^Statistical significance.

### Vulvar assessment and pelvic floor muscle examination: VAMP protocol results

During the BME, the vulvar and perianal areas were viewed, the clitoris gland was fully visible in all patients, and no clitoral adhesions were found (*N* = 0; 0%) in the study cohort.

Pelvic floor muscle assessment according to the four examined areas in the NRS score (VAMP protocol) was low in the asymptomatic population: *V* = 1.18 (SD = 2.04); *A* = 0.04 (SD = 0.37); *M* = 0.72 (SD = 1.49); *p* = 0.83 (SD = 1.58).

One-third (33%) of healthy participants (99/300 women) fulfilled the positive criteria of the VAMP protocol (V/M/P NRS ≥3), suggesting pelvic floor dysfunction (overactivity), which was defined as the VAMP+ group (in contrast to others, defined as the VAMP− group). In the VAMP+ group, the component scores were borderline: *V* = 3.26 (SD = 2.40); *A* = 0.07 (SD = 0.50); *M* = 1.96 (SD = 2.03); *p* = 2.25 (SD = 2.06).

A comparison of the group characteristics with the VAMP protocol examination NRS ≥3: the VAMP+ group is presented in Table 4. Overactive pelvic floor muscle dysfunction (the VAMP+ group) was positively correlated (Chi-square Pearson; significance *p* > 0.05) with backache, anal pain, or other discomforts, dysuria (frequency, urgency, and recurrent lower urinary tract infection), and also vulvar recurrent spontaneous itchiness, soreness, pain, or other vulvar discomforts. Neither dyspareunia nor urinary incontinence was significantly correlated with pelvic floor muscle overactivity (VAMP+ results) in the study cohort.

**Table 4:** Characteristics of the group with the VAMP protocol examination NRS ≥3: VAMP+.

Characteristics	VAMP+ (*p*-Student’s test or Chi-square Pearson): ^*^p < 0.05; ^**^p < 0.01
Age	*p* = 0.422
BMI	*p* = 0.233
Education	*p* = 0.742
Vaginal delivery	*p* = 0.828
Cesarean delivery	*p* = 0.598
Miscarriage	*p* = 0.362
Systolic blood pressure	*p* = 0.894
Diastolic blood pressure	*p* = 0.448
Backache	*p* = 0.037^*^ In the VAMP+ group 53.54% of women In the VAMP – group 40.80% of women
Anal pain or other discomfort	*p* = 0.002^**^ In the VAMP+ group 18.18% of women In the VAMP− group 6.47% of women
Dysuria (frequency, urgency, and recurrent lower urinary tract infection)	*p* = 0.022^*^ In the VAMP+ group 28.28% of women In the VAMP− group 16.92% of women
Urinary incontinence	*p* = 0.189
Vulvar recurrent spontaneous itchiness, soreness, pain, or other discomfort	*p* = 0.002^**^ In the VAMP+ group 26.26% of women In the VAMP− group 12.44% of women
Superficial dyspareunia (vulvovaginal pain or any other discomfort connected to vaginal penetration)	*p* = 0.071 In the VAMP+ group 37.04% of women In the VAMP− group 23.68% of women

VAMP+ group: vulvar., pelvic floor muscles, or periurethral pain during VAMP protocol examination, at least one area (V, M, P) with NRS ≥ 3. VAMP− group: vulvar., pelvic floor muscles, or periurethral pain during protocol VAMP examination of every examined area (V, M, P) with NRS < 3. ^*^Statistical significance.

### Correlation of SQOL and VAMP (both groups)

**Figure 5:**
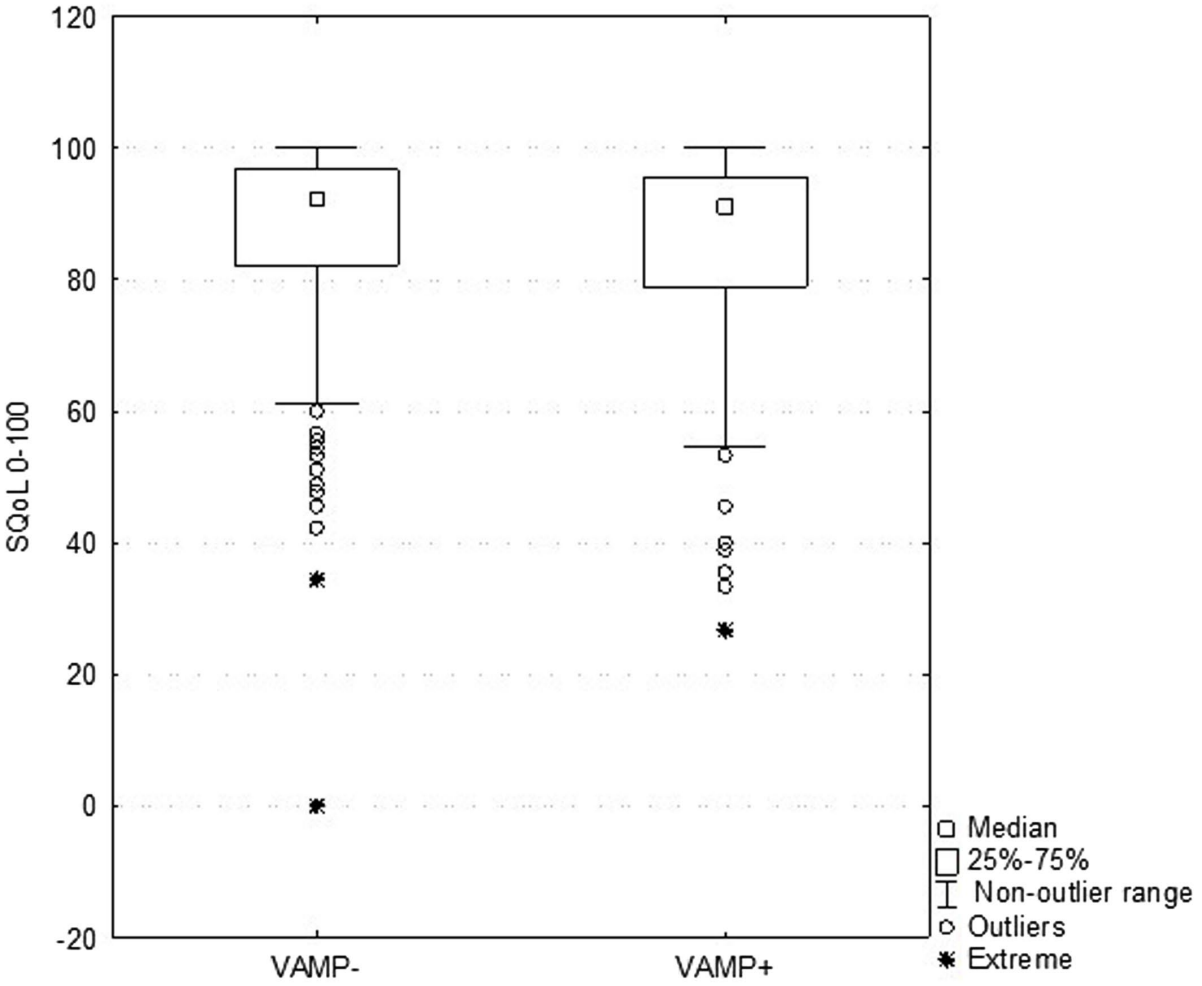
Correlation between sexual quality of life (SQOL) general score and each item in both groups: VAMP+ (with VAMP protocol examination NRS ≥3) and VAMP− (with VAMP protocol examination NRS <3).

An analysis for discriminant validity through a Mann–Whitney test showed that both groups, VAMP+ and VAMP− asymptomatic women, with relation to SQOL total score, did not have a significant difference (VAMP+, *N* = 99, and mean rank = 83.7 and VAMP−, *N* = 210, and mean rank = 86.5), with *Z* = 1.435; *p* = 0.151 (Figure 5).

There was no difference when consecutive items were analyzed separately (Table 5); the correlation was not confirmed.

**Table 5:** Correlation between sexual quality of life (SQOL) VAMP+ and VAMP− groups, each item (18) and the total score (no statistical significance found).

SQOL items	VAMP− (*N* = 210)	VAMP+ (*N* = 99)	Mann–Whitney test: *Z*	Mann–Whitney test: p^*^
1. When I think about my sex life, I feel, I think that generally, it is a pleasant part of my life.	1.5	1.7	−1.511	0.130
2. When I think about my sex life, I feel frustrated.	5.2	5.0	0.647	0.517
3. When I think about my sex life, I feel depressed.	5.5	5.2	1.593	0.111
4. When I think about my sex life, I feel less of a woman.	5.6	5.5	0.776	0.437
5. When I think about my sex life, I feel good with myself.	2.0	2.2	−1.528	0.126
6. I lost confidence in myself as a sexual partner.	5.4	5.2	1.601	0.109
7. When I think about my sex life, I feel anxious.	5.6	5.4	1.091	0.275
8. When I think about my sex life, I feel rage.	5.6	5.5	0.342	0.731
9. When I think about my sex life, I feel closer to my partner.	1.7	1.8	−0.138	0.890
10. I worry about the future of my sex life.	5.0	4.7	0.292	0.769
11. I lost pleasure in sexual intercourse.	5.3	5.3	0.621	0.534
12. When I think about my sex life, I feel embarrassed.	5.4	5.3	0.227	0.820
13. When I think about my sex life, I feel I can speak to my partner about sexual matters.	1.7	1.8	−0.769	0.441
14. I try to avoid sexual intercourse.	5.1	5.1	−0.012	0.989
15. When I think about my sex life, I feel guilty.	5.6	5.5	−0.145	0.884
16. When I think about my sex life, I am scared that my partner will feel hurt or rejected.	5.3	5.1	0.745	0.456
17. When I think about my sex life, I feel as if I have lost something.	5.4	5.3	0.229	0.818
18. When I think about my sex life, I am satisfied with the frequency of sexual intercourse.	2.5	2.8	−1.718	0.085
Total mean score	86.5	83.7	1.435	0.151

^*^As significance level of a value of *p* < 0.05.

## Discussion

In our study result, every asymptomatic woman showed interest in discussing sexual health problems with the gynecologist, even in the “conservative Polish population” (34) which is inconsistent with the literature, where women (and physicians) are “often reluctant to discuss sexual issues” (6). When improving sexual healthcare during a visit to a gynecologist is considered, a targeted strategy should be developed in three specific directions:

1. To find a way to start the discussion with the asymptomatic patients and to enhance the patients’ interest in revealing the sexual health problem to be resolved.
2. Improving the knowledge of sexual health practitioners in order to prevent feelings of shame, disregard, or avoidance, and giving the practitioners enough time for detailed conversations.
3. Gynecologists should know the consecutive steps to refer the patient to another specialist or direct the patient to e-health resources, and reassure the patient that she will not be left without professional support.

The study is mostly dedicated to the first strategy, although other directions are also mentioned.

When asymptomatic patients are asked about their sex life, their answers may be disingenuous, as one-sixth of the study participants denied dyspareunia in face-to-face consultations, but later admitted to it in a tablet survey. Dyspareunia was an exclusion criterion for the study, as dyspareunia is the most common sexual dysfunction and easy to diagnose by asking a simple question, although this was not the case.

At the 2023 annual meeting of the Society of General Internal Medicine, experts stressed that usually patients want to talk about these problems but need their doctors to be ready for such conversations (35), implementing the “5As” framework was recommended:

1. Ask. Start by asking patients if they would be comfortable with you posing a few questions about their sexual health.

In the study, every patient accepted the suggestion.

1. Advise. Make sure your patient knows that many women struggle with the problem they have raised.

In the study, the patient was not reassured of that.

1. Assess. Ask a set of standardized assessment questions.

The questionnaire on the electronic device gave the patient a chance to disclose the problem. For the healthcare practitioner, it can give an opportunity to initiate a discussion about objective test results.

1. Assist. Tell your patient about treatment options.

Clarify concerns; training gynecologists in sexology is required.

1. Arrange. Arrange a follow-up visit to see if treatment has been effective.

Referral to a sex specialist (sex educator, psychologist, psychiatrist, and sexologist, if possible).

Another simple and useful technique practiced by the study authors in regular practice is called the PLISSIT Model: “giving permission (P) for patients to discuss their sexual concerns, providing limited information (LI) and specific suggestions (SS) to help, and then referring for intensive therapy (IT) if needed” (36).

Most patients prefer active questions about sexual health from healthcare professionals; according to the other authors, “physicians should ask all patients about having sexual concerns and that physicians should initiate these conversations. Younger study participants were more likely to have this preference, identified embarrassment as the most common barrier to sexual health conversations. Participants indicated it was easier to discuss sexual concerns with physicians of the same gender and/or a physician they had seen before” (37).

Sexual healthcare on follow-up visits, according to ACOG (38), is usually not implemented by gynecologists. According to the study authors’ statement, the most influential factor in doctor–patient communication about sexuality is the sense of importance of the sexual health problem. The study’s authors are responsible for highlighting this issue in the Polish Gynecologists and Psychiatrist Society.

Based on the study result, it was easier for the patient to reveal the sexual problems without giving face-to-face answers (i.e., answering the SQOL questionnaire and simply asking questions). Patients are not accustomed to disclosing sexual health problems to a gynecologist unless personal or relationship problems become significant.

Using flyers in the waiting room, handing out questionnaires to fill out before an appointment, or sending emails the day before, when the patient has had a chance to think about the questions, should also be the impetus for discussing sexual health issues, with the information that a gynecologist is ready for discussion.

The sexual quality of life questionnaire is one of the broad spectrum of validated tools to be used. Using patient-reported outcomes (PROs) covering sexual function and quality of life before the appointment (on an electronic device, such as a tablet, the results would be available in the office during the patient’s visit) seems to be useful for asking relevant questions if necessary. A review of currently available female sexual dysfunction questionnaires suggests that “although there are numerous instruments, covering sexual function and quality of life, not[*sic*] all were sufficiently comprehensive nor were they applicable to all populations” (39) and not relevant to routine clinical practice, some are allowed to be performed by psychologists or qualified staff. Evidence suggests that “it would facilitate discussion of sexual issues between the clinician and the woman and increase the likelihood of diagnosing sexual dysfunction” (40), as the study authors strongly recommend including questionnaires as part of routine collaboration with women. The study authors note that questionnaires should be used as a part of a general assessment but should not replace a detailed history taking and examination to effectively diagnose and influence coping with sexual problems.

The benefits of a patient’s use of a tablet or smartphone in connection with a gynecological visit have been highlighted by other authors: “the use of new technologies have[*sic*] enabled not only the collection of self-reported measures, but may facilitate the collection of indirect measures” (39, 41) and also facilitates getting quick results instead of manually analyzing, collecting, and utilizing paper-based approaches. In medical practices where PROs are widely used, computerized data analysis systems are essential, which have a number of advantages over paper-based assessments, and there are sufficient data showing that the two methods of administration are equivalent (39, 41), and “complex skip patterns can be programmed or Computer Adaptive Testing (CAT) methods, can be applied to ease administration” (39, 41). Many other modern technologies (apps and wearable devices) are becoming irreversibly part of the gynecologist’s work.

According to the study authors’ experience, the private healthcare sector shows a greater understanding of the complex bio-psycho-social spectrum of sexual problems because healthcare professionals are more flexible, may offer a wider spectrum of education or support, and additional staff is involved to fulfill the patients’ requirements. A center in Poland dedicated to patients with vulvovaginal disease, as Rubin R., a urologist and sex educator (42) has moved patients beyond the traditional clinical model (a 10 min visit covered by insurance companies) to at least 3 h multidisciplinary sexual health evaluation that includes a physical therapist, sexologist, urologist, and others.

The vast majority of women who come for follow-up gynecological visits presented high SQOL (82% of SQOL total score > 84), which is consistent with other authors considering a generally healthy, premenopausal population (29–31).

The four factors extracted from the SQOL could be found in the study; although the subscales were not analyzed, reporting the total SQOL score would be more accurate, especially in a high-scoring population. Similarly, the tool’s developers suggested that “all of the instrument’s intrinsic concepts are strongly interrelated and should be assessed in the overall total score, rather than as separate domains” (12, 43).

This study aimed to improve women’s sexual health by identifying predictors of poor quality of sexual life as well as sexual dysfunction in women of reproductive age. In the study, neither age (from 18 to 50 years) nor BMI were predictors of sexual problems, although according to the literature, quality of sexual life is most impaired in women with Class III obesity (BMI ≥ 40), which was the case for one study participant (44). The contraception methods (non-medical and medical) had no influence on SQOL results. Furthermore, large cross-sectional and longitudinal studies “have not shown a correlation of[*sic*] medical contraceptive methods (hormonal and intrauterine devices) and sexual function in women” (45).

Predisposing factors for sexual dysfunction in women are multidimensional. Identifying potential risk factors helps healthcare professionals modify some of them and determine at-risk populations that require active physician attention to the quality of sexual lives. Understanding the prevalence and determining the risk of sexual dysfunction is also important for prevention efforts. McCool-Myers et al. indicate “significant risk factors for sexual dysfunction among women: poor physical health, poor mental health, stress, abortion, genitourinary problems, female genital mutilation, relationship dissatisfaction, sexual abuse, and religiosity. Important protective factors were older age at marriage, physical activity, daily affection, intimate communication, positive body image, and sex education. Some additional factors had unclear effects: age, education, employment, parity, being in a relationship, frequency of sexual intercourse, race, alcohol consumption, smoking, and masturbation” (46).

Study results indicate that parity, recurrent vulvovaginal symptoms, bladder, anal, and bowel symptoms, or other chronic pain syndromes (low back or joint pain, migraine, temporomandibular junction dysfunction, and gastric symptoms) in otherwise healthy women had no detrimental influence on sexual health. According to other studies, patients with chronic pain (in the reactive arthritis group) limiting social life “had 4.8 times higher risk of sexual dysfunction than other patients” (47).

The psychiatric disorders (and symptoms) group (37% of the study group) had lower sexual wellbeing, although the characteristic did not correlate with a poor/moderate SQOL score (<84 points). Although the mental health problems have not been specified, the results are consistent with those of the other studies (48). Depression and anxiety assessment scales “can be used to identify at-risk individuals who need further evaluation.” Many screening instruments are available in gynecological practice (i.e., the Beck Depression Inventory and the State–Trait Anxiety Inventory), taking into consideration that “the primary health care can overlook up to 50% of patients with depression” (49, 50).

Recommendations for Well-Woman Care (A Well-Woman Chart) was developed by the Women’s Preventive Services Initiative (WPSI), US Preventive Services Task Force (USPSTF), and ACOG (Statement on Depression Screening), a framework for integrating preventive women’s health services into clinical practice: “depression and anxiety screening, using validated questionnaires is recommended annually” (49, 50), also because “women with depression are more likely to suffer from sexual dysfunction than others” (51).

When sexual problems become sexual dysfunction and require treatment, it is not easy for gynecologists to distinguish. A gynecology consultation must determine the appropriate directions to indicate the problem and a referral for professional sexual care. It is crucial “to build a multi-disciplinary and multi-professional network for sexual health. This network should provide professional state-of-the-art patient management and care in case of sexual health issues, problems, dysfunction, or need for sex-education[*sic*], sexual medicine, and sexual therapy” (52).

Data from some studies have shown an increased need for routine treatment and the management of sexual healthcare as a team with a psychiatrist and psychotherapist, which is increasingly important in gynecological practice. These data underscore “the need for professional qualification in sexual medicine and expanded collaboration between different medical disciplines and healthcare professionals to integrate sexual health topics into daily routine” (52).

According to the literature, “pelvic floor muscles has been found overactive in 70–80% of women with bladder, bowel, and sexual disorders” (53). Genitourinary pain symptoms were assessed in correlation with a bimanual pelvic exam (VAMP protocol) during a well-woman’s visit. The hypothesis of the study authors was not confirmed, and pelvic floor muscle overactivity (elevated VAMP score) was not a predictor of poor quality of sexual life in asymptomatic women. In the study group, there was no correlation between SQOL score and VAMP+ (NRS ≥3), which indicates that pelvic floor muscle assessment during a well-woman visit is not mandatory. In the study population, only a correlation between worse sexual wellbeing and dyspareunia was found. Patients with dyspareunia have a more than three times higher chance of having poor or moderate sexual life when compared with non-dyspareunia women. Dyspareunia, as pain associated with sexual activity (mainly during penetration), is a sexual dysfunction that affects 10–20% of women (54) and is perceived as the strongest factor in lower SQOL (55). According to the literature, “endometriosis worsen[*sic*] SQOL what[*sic*] was significantly and independently associated with the presence of deep dyspareunia, as the pelvic pain during penetration” (56).

Superficial dyspareunia is most often caused by inappropriate foreplay, violent sex, and provoked vulvodynia. Vulvodynia in 80–90% of cases is associated with PFMD/overactivity (57), which is also the cause of other comorbid diseases. One-third of asymptomatic women scored positive on the VAMP scale (VAMP+), although in the vast majority of cases, the score was borderline. The estimated prevalence of pelvic floor myofascial pain (upon examination) ranges from 14 to 78% and is the highest in studies where a common assessment is performed. An underappreciated area contributing to sexual health and sexual dysfunction is pelvic floor muscle overactivity (53). The low increase in the overactive state in the study cohort (the VAMP+ group) more often complained of backache, anal discomfort or pain, dysuria (frequency, urgency, and recurrent lower urinary tract infection), and vulvar recurrent spontaneous itchiness, soreness, pain, or other discomfort but was not correlated with urinary incontinence or superficial dyspareunia. Another study confirmed a high VAMP score in women with provoked vulvodynia (as the cause of superficial dyspareunia) (26). The results of the present study indicate that the low increase in cotton swab vestibular pain (V in the VAMP protocol) and pelvic floor muscle pain (M and P in the VAMP protocol) are not sufficient to suspect sexual dysfunction in asymptomatic women. The results were consistent with those of other authors, where “pain upon palpation of the pelvic floor muscle in asymptomatic, nulliparous women should be considered an uncommon finding” (58).

Whether asymptomatic women benefit from a routine pelvic exam (BPE) has recently been questioned. A gynecological exam helps to clarify symptoms that the patient downplayed or rule out abnormalities if the patient had concerns. One study of adult women (aged 21–65 years) found that “62% of respondents felt that the bimanual examination helped them establish open communication with their gynecologist, and 82% felt that the examination provided them with reassurance about their condition” (59), so it should be maintained in well-woman visits.

### Limitations of the study

The survey results should be interpreted with several limitations. Answering the questions (after the visit) on the tablet, took the patient additional time (survey and questionnaire), which may have affected the reliability of the data. Filling out a short questionnaire before the appointment with comments during the follow-up visit seems to have an advantage. The questionnaire requires participants to provide intimate information, which may not be comfortable enough to address with any accuracy. The SQOL questionnaire is usually performed along with other validated tests to examine the quality of life with the coexistence of specific sexual dysfunctions or other health problems that have not been evaluated in the current study. Using an online questionnaire, the sensitive data were collected anonymously in the research, although when the result is used at the visit, the patient should provide the data, which may not be convenient and requires strict data protection.

### Implications for clinical practice

Although ACOG published the Well-Woman Task Force Report supporting the elements of well-woman screening with expert consensus from 15 major women’s health organizations, it provided the credentials needed for healthcare providers to further update their practices. Recommended by the Well-Woman Task Force, the screening elements for healthy, non-pregnant adult women of childbearing age are based on 29 elements, divided into 6 groups (8), which show the directions for gynecological practice, although they appear to be complicated.

Finding a way to start discussions with asymptomatic patients is key to increasing patients’ interest in disclosing a sexual health problem that needs to be addressed, mainly among those at risk for worsening sexual wellbeing.

PRO measurements or simple questions about sexual wellbeing before a visit, on an electronic device, to discuss sexual health with patients, are useful, mainly in the at-risk group for sexual deterioration: those with mental health problems and women with dyspareunia.

Pelvic floor muscle assessment during a well-woman visit is not mandatory. Once evidence-based, standardized principles for conducting a healthy woman’s visit have been developed, efforts can be directed toward promoting the education of gynecologists.

There is a need to engage new technologies in the course or preparation for a gynecological visit (or between follow-up consultations) to avoid moving gynecological prevention exclusively into the realm of the Internet.

### Further research and publications

An indication for further research and publication is the preparation of reliable and clear guidelines and training for gynecologists on how to support the quality of sexual life in asymptomatic women during the follow-up visit so that the opportunity for an evidence-based medical approach is not missed. Otherwise, patients and their partners will use other sources of information, and healthcare practitioner significance will be marginalized. “Present sexual health education for students and practicing physicians is inadequate to meet advances in science and technology and growing patient demand for quality sexual health care. There is a need for better training at medical institutions responsible for training doctors in sexual health worldwide” (60, 61). Therefore, there is a need to develop new training programs based on expert knowledge and previous reports and conduct further studies to evaluate the impact of activities on improving the sexual wellbeing of female patients. Education programs at various stages should be evaluated in terms of skill acquisition and the implementation of knowledge into clinical practice, just as they have been developed in the field of urinary incontinence (62).

There is an urgent need to actively engage in improving women’s health around the world, and it is imperative that it becomes a priority for policymakers and politicians. This requires collaboration with key stakeholders, including women, caregivers, researchers, healthcare providers, and non-governmental organizations, because “it’s time to prioritize women’s health” (63).

## Conclusion

Women’s sexual health is a global health priority. Finding a way to start a discussion with an asymptomatic patient is crucial to increasing patients’ interest in disclosing a sexual health problem to be resolved. PRO measurements prior to the visit, on an electronic device, to discuss sexual health issues with patients are useful. PROs or simple questions about sexual wellbeing direct the discussion mainly toward the at-risk group for sexual deterioration: those with mental health problems and women with dyspareunia. Another study hypothesis was not confirmed: overactivity of the pelvic floor muscles (VAMP+) was not a predictor of poor quality of sexual life, and the assessment of the pelvic floor in asymptomatic women is not mandatory. Dyspareunia is considered a predictor of decreased quality of sexual life, a main sexual dysfunction that should not be overlooked. Gynecological consultation should resolve sexual wellbeing concerns, identify the problem, and refer for professional sexual care if still needed.

## Data availability statement

The original contributions presented in the study are included in the article/supplementary material, further inquiries can be directed to the corresponding author.

## Ethics statement

Ethical approval was not required for the study involving humans in accordance with the local legislation and institutional requirements. Written informed consent to participate in this study was not required from the participants in accordance with the national legislation and the institutional requirements.

## Author contributions

EB-R: Conceptualization, Data curation, Formal analysis, Investigation, Methodology, Writing – original draft. JW-B: Data curation, Investigation, Project administration, Software, Visualization, Writing – original draft. TP: Formal analysis, Funding acquisition, Supervision, Writing – review & editing.

## Footnotes

^1^https://profitest.pl/
